# Bioactivities of major constituents isolated from *Angelica sinensis *(*Danggui*)

**DOI:** 10.1186/1749-8546-6-29

**Published:** 2011-08-19

**Authors:** Wen-Wan Chao, Bi-Fong Lin

**Affiliations:** 1 Department of Biochemical Science and Technology, College of Life Science, National Taiwan University, Taipei 10617, Taiwan

## Abstract

*Danggui*, also known as *Angelica sinensis *(Oliv.) Diels (Apiaceae), has been used in Chinese medicine to treat menstrual disorders. Over 70 compounds have been isolated and identified from *Danggui*. The main chemical constituents of *Angelica *roots include ferulic acid, Z-ligustilide, butylidenephthalide and various polysaccharides. Among these compounds, ferulic acid exhibits many bioactivities especially anti-inflammatory and immunostimulatory effects; Z-ligustilide exerts anti-inflammatory, anti-cancer, neuroprotective and anti-hepatotoxic effects; n-butylidenephthalide exerts anti-inflammatory, anti-cancer and anti-cardiovascular effects.

## Background

**Figure 1:**
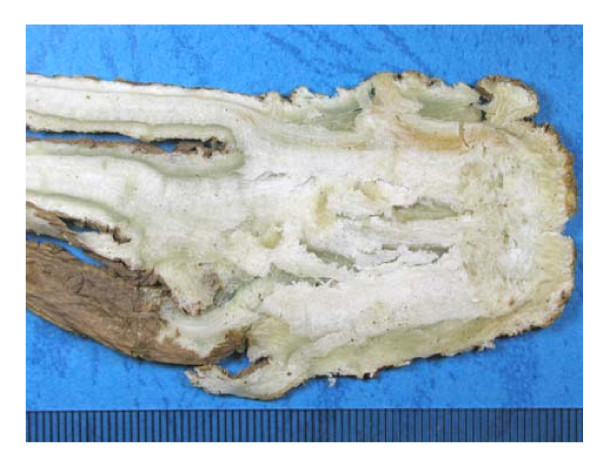
**A section of root of *Angelica sinensis *(Oliv.) Diels (Apiaceae) used in Chinese medicine**.

*Angelica sinensis *(Oliv.) Diels (Apiaceae) (AS), the root of which is known in Chinese as *Danggui *(Figure 1), was first documented in *Shennong Bencao Jing *(*Shennong's Materia Medica*; 200-300AD) and has been used as a blood tonic to treat menstrual disorders [1]. *Danggui *is marketed in various forms worldwide [2,3]. Over 70 compounds have been identified from *Danggui*, including essential oils such as ligustilide, butylphthalide and senkyunolide A, phthalide dimers, organic acids and their esters such as ferulic acid, coniferyl ferulate, polyacetylenes, vitamins and amino acids. Z-ligustilide (water insoluble and heat stable), among which Z-butylidenephthalide and ferulic acid are thought to be the most biologically active components in AS [4] and are often used in quality control and pharmacokinetic studies of *Danggui *[3-6].

Z-ligustilide is the main lipophilic component of the essential oil constituents and a characteristic phthalide component of a number of Umbelliferae plants. Z-ligustilide is considered to be the main active ingredient of many medicinal plants, such as *Danggui *[7] and *Ligusticum chuangxiong *[8].

### Phthalides

**Figure 2:**
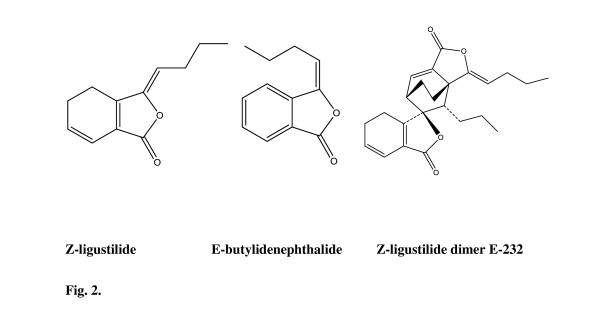
**Chemical structures of various identified phthalides found in *Angelica sinensis***.

Phthalides (Figure 2) consist of monomeric phthalides such as Z-ligustilide and phthalide dimers. In 1990 *Danggui *was reported in the literature when the Z-ligustilide dimer E-232 was isolated [9]. The majority of the phthalides identified is relatively non-polar, the fraction of which can be extracted with solvents such as hexanes, pentane, petroleum ether, methanol, 70% ethanol and dichloromethane. The amount of Z-ligustilide in *Danggui *varies between 1.26 and 37.7 mg/g dry weight [6,10,11]. Z-ligustilide facilitates blood circulation, penetrates the blood brain barrier to limit ischemic brain damage in rats and attenuates pain behaviour in mice [12-14]. Preclinical studies have indicated that AS and Z-ligustilide may also relax smooth muscle in the circulatory, respiratory and gastrointestinal systems [15].

### Organic acids

**Figure 3:**
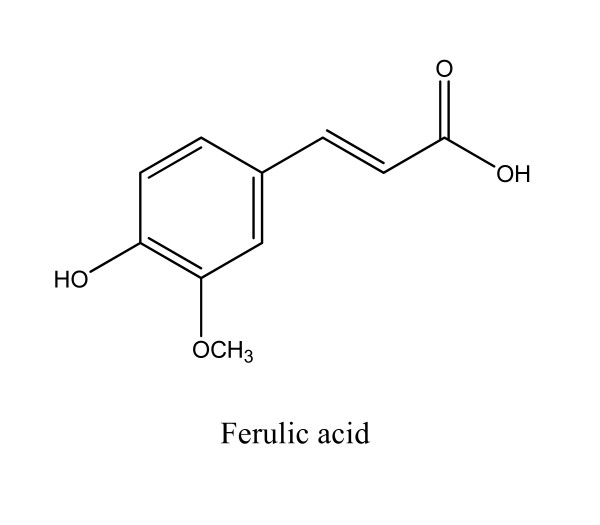
**Chemical structure of the major organic acid in *Angelica sinensis***.

*Danggui *contains many organic acids. For example, ferulic acid (Figure 3) isolated from *Danggui *is widely used as the marker compound for assessing the quality of *Danggui *and its products. Methanol, methanol-formic acid (95:5), 70% methanol, 70% ethanol, 50% ethanol or diethyl ether-methanol (20:1) is used as the initial extraction solvent. The amount of ferulic acid in *Danggui *varies between 0.21 and 1.75 mg/g dry weight [6,16]. We recently extracted ferulic acid from AS using ethyl acetate and obtained 3.75 mg/g dry weight of the whole plant [11]. Abundant in rice bran, wheat, barley, tomato, sweet corn and toasted coffee, ferulic acid is an antioxidant, anti-inflammatory and anti-cancer agent and apart from its effects against Alzheimer's disease, it possesses anti-hyperlipidemic, antimicrobial and anti-carcinogenic properties [17-21].

### Polysaccharides

Biochemical and medical researchers have recently been interested in the anti-tumor and immunomodulatory effects of polysaccharides [22]. The efficacy of *Danggui *is associated with its various polysaccharides [22] which are extracted with water as the initial extraction solvent. Polysaccharides from *Danggui *consist of fucose, galactose, glucose, arabinose, rhamnose and xylose [23]. *Danggui *contains a neutral polysaccharide and two kinds of acidic polysaccharides [24].

## Pharmacological activities

### Anti-inflammatory effects

Ferulic acid and isoferulic acid inhibit macrophage inflammatory protein-2 (MIP-2) production by murine macropharge RAW 264.7 cells, suggesting that these compounds contribute to the anti-inflammatory activity of AS [25,26]. Z-ligustilide also shows anti-inflammatory effects, probably related to inhibition of the TNF-α and NF-κB activities [27]. Using an NF-κB-dependent trans-activation assay as a pre-screening tool, our study demonstrated the anti-inflammatory effect of the ethyl acetate fraction of AS or *Danggui *[28]. AS suppresses NF-κB luciferase activity and decreases NO and PGE_2 _production in lipopolysaccharide (LPS)/IFN-γ-stimulated murine primary peritoneal macrophages. Ferulic acid and Z-ligustilide, two major compounds in AS, decrease NF-κB luciferase activity, which may contribute to the anti-inflammatory activity of AS [11]. Our *in vivo *study further confirmed that the ethyl acetate extract of AS inhibits the production of inflammatory mediators thereby alleviating acute inflammatory hazards and protecting mice from endotoxic shock [29]. Using a murine air pouch model, Jung *et al*. reported that the leukocyte count in the pouch exudate decreases in the *BALB/c *mice fed with 100 mg/kg body weight of a root extract (*A. senticosus*: *AS*: *Scutellaria baicalensis*), accompanied by a decrease in the neutrophil count, IL-6 mRNA level and TNF-α mRNA level in the pouch membrane and by decreased IL-6 and PGE_2 _concentrations in the pouch fluid and that the concentration of anti-inflammatory PGD_2 _in the pouch fluid increases as well [30]. Fu *et al*. reported that n-butylidenephthalide decreases the secretion of IL-6 and TNF-α during LPS stimulated activation of murine dendritic cells 2.4 *via *the suppression of the NF-κB dependent pathways [31].

### Anti-cancer effects

AS extract induces apoptosis and causes cell cycle arrest at G_0_/G_1 _in brain tumor cell lines [32]. AS extract also decreases the expression of the angiogenic factor vascular endothelial growth factor (VEGF) in brain astrocytoma [33]. Moreover, n-butylidenephthalide and Z-ligustilide are cytotoxic against brain tumor cell lines [34] and leukemia cells [35]. The three main AS phthalides, namely n-butylidenephthalide, senkyunolide A and Z-ligustilide, decrease cell viability of colon cancer HT-29 cells dose-dependently [36]. Yu *et al*. reported that pretreatment of the PC12 cells with Z-ligustilide attenuates H_2_O_2_-induced cell death, attenuates an increase in intracellular reactive oxygen species (ROS) level, decreases Bax expression and cleaves caspase-3 and cytochrome C [37]. A novel polysaccharide (50 mg/kg, 100 mg/kg) isolated from AS inhibits the growth of HeLa cells in nude mice *via *an increased activity in the caspase-9, caspase-3 and poly (ADP-ribose) polymerase (PARP) [38].

### Immunomodulatory effect

Treatment of *BALB/c *mice spleen cells with AS polysaccharide (100 μg/ml) increases the production of IL-2 and IFN-γ and decreases the production of IL-4 [39]. An acidic polysaccharide fraction isolated from AS stimulates female *BALB/c *murine peritoneal macrophages to produce higher levels of nitric oxide (NO) *via *the induction of iNOS gene expression [40]. The AS polysaccharide (mannose, rhamnose, glucuronic acid, galacturonic acid, glucose, galactose, arabinose, xylose)-dexamethasone conjugate demonstrates a therapeutic effect on trinitrobenzenesulfonic acid-induced ulcerative colitis in rats and the systemic immunosuppression caused by dexamethasone [41]. Four hydrosoluble fractions of AS polysaccharide exert the most conspicuous mitogenic effects on phagocytic activity and NO production by female ICR mouse peritoneal macrophages [42]. AS polysaccharide treatment rescues *BALB/c *mice from retro-orbital bleeding induced anemia and increases IL-6, granulocyte-macrophages colony stimulating factor (GM-CSF) concentrations in spleen cells [43].

Ferulic acid, an antioxidant from AS, decreases H_2_O_2_-induced IL-1β, TNF-α, matrix metalloproteinase-1 and matrix metalloproteinase-13 levels and increases SRY-related high mobility group-box gene 9 gene expression in chondrocytes [44]. AS induces the proliferation of ICR murine bone marrow mononuclear cells by activating ERK1/2 and P38 MAPK proteins [45]. Pretreatment with 50 mg/kg AS increases serum colony-stimulating activity together with IFN-γ and TNF-α levels in the spleen mononuclear cells of *Listeria monocytogenes*-infected *BALB/c *mice [46].

### Anti-cardiovascular effects

Pre-treatment with AS (15 g/kg daily for 4 weeks) decreases doxorubicin-induced (15 mg/kg intravenously) myocardial damage and serum aspartate aminotransferase levels in male ICR mice [47]. Human umbilical vein endothelial cells (HUVECs) treated with AS water extract activate VEGF gene expression and the p38 pathway, thereby increasing angiogenic effects of HUVECs both *in vitro *and *in vivo *[48].

Excess adipose tissue can lead to insulin resistance and increases the risk of type II diabetes and cardiovascular diseases. Water and 95% ethanol extracts of AS effectively decrease fat accumulation in 3T3-L1 adipocytes and reduce triglyceride content [49]. Yeh *et al*. demonstrated that n-butylidenephthalide is anti-angiogenic and is associated with the activation of the p38 and ERK1/2 signaling pathways [50].

### Neuroprotective effects

Z-ligustilide treatment decreases the level of malondialdehyde (MDA) and increases the activities of the antioxidant enzymes glutathione peroxidise (GSH-Px) and superoxide dismutase (SOD) in the ischemic brain tissues in ICR mice; meanwhile there is a decrease in Bax and caspase-3 protein expression [51]. Z-ligustilide increases the choline acetyltransferase activity and inhibits the acetylcholine esterase activity in ischemic brain tissue from Wistar rats [52]. Huang *et al*. reported that AS extract protects Neuro 2A cell viability against β-amyloid (Aβ) peptide induced oxidative damage by ROS, MDA and glutathione (GSH) and rescues mitochondrial transmembrane potential levels [53]. Z-ligustilide inhibits the TNF-α-activated NF-κB signaling pathway, which may contribute to Z-ligustilide's protective effect against Aβ peptide-induced neurotoxicity in rats [54].

AS methanol extract significantly attenuates Aβ_1-42 _induced neurotoxicity and tau hyperphosphorylation in primary cortical neurons [55]. AS polysaccharides (18.6% saccharose) reduce myocardial infarction size and enhance cardiotrophon-1 levels, serum GSH levels, serum SOD levels, GSH-Px activity and brain caspase-12 expression in Wistar rats treated with a single oral dose (100, 200, 300 mg/kg) daily for two months [56].

A multi-herbal mixture composed of *Panax ginseng*, *Acanthopanax senitcosus*, AS and *S. Baicalensis*, HT008-1 down regulates COX-2 and OX-42 expression in the penumbra region [57].

### Anti-oxidative activities

Water AS extract can be further purified into various AS polysaccharide fractions, such as a highly acidic polysaccharide fraction consisting of galacturonic acid. *BALB/c *murine peritoneal macrophages pretreated with various AS polysaccharide fractions alleviate the decrease in cell survival caused by *tert*-butylhydroperoxide, with an increased intracellular GSH content [58]. Furthermore, acidic polysaccharide fraction is also the most active fraction in terms of inhibiting the decrease in cell viability caused by H_2_O_2_. Acidic polysaccharide fraction also decreases the MDA formation, reduces the decline in SOD activity and inhibits the depletion of GSH in murine peritoneal macrophages caused by H_2_O_2 _[59]. Ethanol extract of AS combined with eight other Chinese herbs significantly increase the radical scavenging ability of 1,1-diphenyl-2-picryl hydrazine (DPPH) [60].

### Anti-hepatotoxic effects

The liver contains a series of microsome hemoproteins called cytochrome P450s (CYPs). CYPs play an important role in the metabolic oxygenation of a variety of lipophilic chemicals including drugs, pesticides, food additives and environmental pollutants. The most important isoenzyme forms of cytochrome are CYP1A2 (13%), CYP2C (20%), CYP2D6 (2%), CYP2E1 (7%) and CYP3A (29%) [61]. Tang *et al*. reported that water and ethanol extracts of AS strongly increase the CYP2D6 and CYP3A activity in the microsome fraction of male Wistar rat livers [62]. Gao *et al*. demonstrated that treatment with *Danggui Buxue Tang *(DBT), which contains the roots of *Astragali *and AS, induces erythropoietin mRNA expression in a dose-dependent manner in human hepatocellular carcinoma cell line Hep3B [63]. Dietz *et al*. reported that Z-ligustilide targets cysteine residues in human Keap1 protein thereby activating Nrf2 and the transcription of antioxidant response element (ARE) regulated genes and inducing NADPH:quinine oxidoreductase 1 (NQO1) [64].

### Kidney protective effects

There has been a 60% increase in the number of people needing treatment for chronic kidney disease between 2001 and 2010. Characterized by an increase in interstitial fibrosis and tubular epithelial cell atrophy, renal tubulointerstitial fibrosis is the common pathogenetic process of chronic kidney disease [65-67]. Angiotensin II appears to play a key role in several mechanisms involved in tubulointerstitial fibrosis. Angiotensin II up-regulates the expression of TGFβ1, a profibrotic cytokine involved in many of the events leading to renal fibrosis. Angiotensin converting enzyme inhibitors (ACEi) can reduce renal tubulointerstitial fibrosis [68].

Oral administration of *Astragalus membranaceus *var. *mongholicus *and AS (14 g/kg/day) to Wistar rats increases the constitutive ROS activity in the kidneys. The treatment also enhances NO production *via *eNOS activation and the scavenging of ROS in the obstructed kidney in Wistar rats after unilateral ureteral obstruction [69]. In analysis with genechips, gene expression is induced, including transient receptor protein 3 (TRP3), bone marrow stromal cell antigen 1 (BST-1), peroxisomal biogenesis factor 6 (PEX6), xanthine dehydrogenase (XDH), CYP1A1, serine/cysteine proteinase inhibitor clade E member 1 (PAI-1) and fibroblast growth factor 23 (FGF23). These genes may be involved in the increased degeneration of the extracellular matrix (ECM), decreasing ROS and regulating calcium phosphate metabolism [70]. Administration of *Astragalus membranaceus *var. *mongholicus *and AS into Sprague Dawley rats, as a unilateral ureteral obstruction model, decreases TGFβ1 levels, fibroblast activation, macrophage accumulation and tubular cell apoptosis [71]. Song *et al*. reported that oral administration of *Astragalus membranaceus *var. *mongholicus *and AS (12 g/kg/day) shows renoprotective effects, possibly associated with a reduction of proteinuria and up regulation of VEGF. These changes may have reduced the loss of capillaries and improve microstructure dysfunction in nephrectomized rats [72].

A study on 47 herbs of potential interest in the context of renal or urinary tract pathologies demonstrated that AS, *Centella asiatica*, *Glycyrrhiza glabra*, *Scutellaria lateriflora *and *Olea europaea *have strong antioxidant effects in tubular epithelial cells or apoptotic effects on renal mammalian fibroblasts or both [73].

### Other effects

Ethanol extract of AS (100, 300 mg/kg) prolongs estrus in rats and the estrogenic activity of AS extract is likely due to the presence of Z-ligustilide [74]. A dichloromethane extract of AS exhibits a potent inhibitory effect on melanin production [75]. AS polysaccharide (ASP, 2.5 mg/day) enhances recovery in platelet, red blood cells and white blood cells counts in *BALB/c *mice after irradiation (4 Gy) [76]. ASP also reduces hepcidin expression by inhibiting signal transducer and activator of transcription (STAT) 3/5 and decapentaplegic protein (SMAD) 4 expression in the liver. Thus, ASP is suggested to be used for treating hepcidin-induced diseases [77].

## Conclusion

Bioactive components extracted from AS roots include Z-ligustilide, ferulic acid and AS polysaccharides. Major pharmacological effects of *Danggui *extract or its components include anti-inflammatory, anti-cancer, immunomodulatory, anti-cardiovascular, neuroprotective, anti-oxidative, anti-hepatotoxic and renoprotective activities.

## Abbreviations

AS: *Angelica sinensis*; MIP-2: macrophage inflammatory protein-2; LPS: lipopolysaccharide; VEGF: vascular endothelial growth factor; ROS: reactive oxygen species; JNK: c-Jun NH_2_-terminal kinase; AP-1: activating protein-1; PARP: poly (ADP-ribose) polymerase; GM-CSF: granulocyte-macrophages colony stimulating factor; ERK1/2: extracellular signal-regulated kinase1/2; HUVEC: human umbilical vein endothelial cell; MDA: malondialdehyde; GSH-Px: glutathione peroxidise; SOD: superoxide dismutase; GSH: glutathione; DPPH: 1,1-diphenyl-2-picryl hydrazine; CYP: cytochrome P450; Nrf2: nuclear factor E2-related factor 2; ARE: transcription of antioxidant response element; ACEi: angiotensin converting enzyme inhibitors; TRP3: transient receptor protein 3; BST-1: bone marrow stromal cell antigen 1; PEX6: peroxisomal biogenesis factor 6; XDH: xanthine dehydrogenase; CYP1A1: cytochrome P450 subfamily I member A1; PAI-1: serine/cysteine proteinase inhibitor clade E member 1; FGF23: fibroblast growth factor 23; STAT3/5: signal transducer and activator of transcription 3/5.

## Competing interests

The authors declare that they have no competing interests.

## Authors' contributions

WWC and BFL searched the literature and wrote the manuscript. Both authors read and approved the final version of the manuscript.
